# Detection of Parvalbumin Fish Allergen in Canned Tuna by Real-Time PCR Driven by Tuna Species and Can-Filling Medium

**DOI:** 10.3390/molecules27175674

**Published:** 2022-09-02

**Authors:** Elif Tugce Aksun Tümerkan

**Affiliations:** 1 Department of Food Processing-Food Technology, Vocational School of Health Services, Ankara Yıldırım Beyazıt University, Ankara 06760, Turkey; etaksun.tumerkan@aybu.edu.tr; 2 AYBU Central Research Laboratory, Application and Research Center, Ankara Yıldırım Beyazıt University, Ankara 06010, Turkey

**Keywords:** allergen, calcium content, canned tuna, filling medium, parvalbumin, real-time-PCR, acidity

## Abstract

Canned tuna is considered one of the most popular and most commonly consumed products in the seafood market, globally. However, in past decades, fish allergens have been detected as the main concern regarding food safety in these seafood products and are listed as the top eight food allergies. In the group of fish allergens, parvalbumin is the most common. As a thermally stable and calcium-binding protein, parvalbumin can be easily altered with changing the food matrices. This study investigated the effect of a can-filling medium (tomato sauce, spices, and brine solutions) on the parvalbumin levels in canned tuna. The effect of pH, calcium content, and the DNA quality of canned tuna was also investigated before the parvalbumin-specific encoded gene amplification. The presence of fish allergens was determined by melting curve analyses and confirmed by agarose gel electrophoresis. The obtained results showed that the presence of parvalbumin in commercially canned tuna was driven by can-filling mediums, thermal conductivity, calcium content, and the acidity of various ingredients in food matrices. The intra-specific differences revealed a variation in fish allergens that are caused by cryptic species. This study proved that allergens encoding gene analyses by agarose electrophoresis could be used as a reliable approach for other food-borne allergens in complex food matrices.

## 1. Introduction

Due to the nutritional benefits, high dietary proteins and high content of healthy lipids, seafood products are widely accepted and preferred in the human diet, globally [1]. Canned tuna is considered to be one of the most traded seafood products globally, due to its long shelf life, transporting benefits, ready-to-eat form, as well as due to the variety of meals that can be prepared with it [2,3]. Despite all of the benefits, the consumption of fish meals cause approximately 90% of total life-threatening allergic reactions, gastrointestinal problems, and respiratory symptoms [4]. Therefore, the occurrence of fish allergies among consumers is one of the main concerns among seafood producers, as well as its prevalence rate of 8% among fish-processing employees [5].

In the groups of fish allergens, a small, conserved muscle protein, the parvalbumin (10–13 kDa, pI 4.1–5.2), is the most common cause of allergic reactions. The parvalbumin is characterized by easy calcium-binding, high stability during thermal food processing, and enzymatic digestion [6,7]. The presence and content of parvalbumin differ from species to species and even different parts of fish body can contain this allergen at different levels. The symptoms and allergenicity that are caused by fish allergens differ depending on inter-specific and intra-specific characteristics that are present [8]. For example, a study conducted by Mourad and Bahna [9] revealed that dark muscle (cartilaginous fish muscle) can contain a higher level of alpha-parvalbumin (non-allergenic protein) than beta-homolog (allergenic protein) does, while the white tissues (bony fish muscle) of the same fish contains a higher level of beta-parvalbumin than alpha-parvalbumin does.

Tuna species, as an umbrella species, are the large group of important fishes that belong to the Scombridae family. The Scombridae family is classified into three genera: Katsuwonus, Sarda, and Euthynnus. All of these species have different economic and ecological values [10]. Although tuna species are considered less allergenic than other often consumed fish species, such as salmon and herring [11,12], the occurrence of parvalbumin is reported in both fresh and processed tuna [13,14]. Due to Blickem et al. [15], undeclared tuna allergen was classified as one of the primary reasons for commercial tuna recalls between 2002 to 2020 in the United States.

Studies shown that the parvalbumin content in canned tuna varies depending on the processing techniques that are used. Thermal processing causes an alternation in the protein solubility and detectability, especially in fish species. Comparing the parvalbumin content in raw and in canned fish, a decrease of 25% parvalbumin concentration was noted after the canning process [7,8]. On the other side, Liang et al. [7] reported that the IgE reactivity of parvalbumin increased after the fish was thermally treated. The differences in the structural homology of parvalbumin that are driven by heating have also been confirmed by the presence of monomeric and oligomeric parvalbumin in thermally processed fish samples [5].

Fishes belonging to the Scombridae family are characterized by high levels of histidine which can be easily converted to histamine by thermal processing during the canning process. Therefore, the consumption of canned tuna can cause the occurrence of histamine-originated allergy-like symptoms in sensitive individuals [16].

The allergenicity of food can be changed with structural changes of the food matrix [17,18]. In the case of canned tuna, allergenicity can be fortified by using a can-filling medium. To expand, as with other types of canned fishes, canned tuna that is placed on the market is combined with different can-filling mediums such as brine solutions, different types of oils, vegetables, and more recently various sauces and ingredients. The pH and/or thermal conductivity of these food matrices are responsible for the alteration of the main food items’ allergenicity.

For instance, the addiction of vinegar has decreased allergenicity due to the acidic denaturation of the allergen-causing proteins [19], while the increased fat content or the presence of lipids in the food matrix can contribute to the stability of the allergenic proteins [20]. Pekar et al. [21] reported that salt and carbohydrate content in a food matrix also can influence on the allergenicity of mixed foods. Jiang and Rao [22] also reported a relation between antigenicity and calcium level in the food matrices as the consequence of parvalbumin being a calcium-binding protein.

No mechanism is known for population’s protection against food allergy occurrences and the avoidance of consumption of the allergen-containing food product is the only prevention method, while a determination of the food-borne allergies that are present in food products can reduce their occurrence among the population [22]. Within national and international regulations that are related to the labeling of the allergen-causing compounds on food products, the importance of the detection of allergens has been well understood for public health safety over the past years.

Protein-based and immunologic analytical methods have been used for the determination of food-borne allergies over the years, but in recent years, researchers have reported DNA barcoding, and especially DNA mini-barcoding, as a promising species authentication method which provides reliable species identification in processed samples within the shorter base phase [23]. DNA-based techniques have been especially preferable in studies for which a processed food products’ allergenicity should be analyzed. For example, a heat treatment, a variation in acidity, or pressure applications can cause the degradation of protein which reduce the recovery of the protein-based methods, while there is stability for the DNA to communicate the sensitivity and reproducibility of the allergens that are detection in the processed food products [24].

Variations in protein molecular weight fragments and the loss of significant definable protein bands on SDS-PAGE have been reported in canned food products [23].

Since canned tuna items are one of the most widely consumed seafood products globally, the detection of any risk to human health that is associated with the consumption of these products is essential to the global food security chain [24]. In the light of this, in the present study, DNA barcoding and DNA mini barcoding has used in this investigation, and the study has focused on: (i) the impact of the acidity and calcium levels of canned tuna samples on the parvalbumin concentration, (ii) the effect that filling has on the parvalbumin concentration, and (iii) the possible use of filling substitutions in order to reduce the presence of the fish allergens in canned tuna products. Within these aims, any potential reasons causing the presence of fish allergens originating from fish muscle and the use of can filling mediums in commercially canned tuna were also investigated.

## 2. Materials and Methods

### 2.1. Materials

The type of oil, sauce, or other ingredients are classified as part of the filling medium group. The major fish allergen, the parvalbumin target gene region, was used for the sensitive detection of allergens in commercially canned tuna products by using a real-time PCR. A total of 29 canned tuna products from 13 different brands representing the most commonly consumed tuna products in Turkey were studied. Canned tuna samples, purchased from local supermarkets, included different filling mediums; tuna in sunflower oil, tuna in olive oil, spiced tuna, and light tuna with different sauces (detailed information is reported in Table 1). All canned tuna samples were largely within their expiry dates, which ranged between October 2024 and April 2026. Subsequently, a DNA-based allergen analysis was carried out in early 2022 on the products that were fully within their shelf-life validity.

### 2.2. pH Measurements of Tuna and Filling Mediums

The pH of the canned tuna samples and filling mediums were measured using a calibrated 315i/SET pH-meter (Weilheim, Germany), as described by Mohan [25]. Spices, oils, and sauces were removed from the tuna flesh using a filter paper and then homogenized in distilled water (1:2 *w*/*v*) using a homogenizer (Ika- Werke Ultra-turrax, Staufen, Germany) at 12,000 rpm for 3 min. Then the pH was measured using a digital pH meter (315i/SET, Weilheim, Germany), as described in IS 2168 (1971). The can-filling mediums were filtered through Whatman filter paper (Whatman Grade No: 4, Whatman, Inc., Florham Park, NJ, USA) and then the electrode of the pH meter was inserted into the filtered mixture.

### 2.3. Calcium Content of Canned Tuna

The calcium content was analyzed using a Perkin-Elmer 700 atomic absorption spectrophotometer (Perkin Elmer Corporation, Norwalk, CT, USA), according to the AOAC method 985.35. An amount of 0.2 g grounded tuna sample was dried in an oven at 100 °C. Following this, the aliquot was dried, and the sample was then placed in 525 °C muffle furnace until the ignition was completed. The collected ash was then dissolved in 5 mL of 1 M nitric acid within the thermal treatment stage. The mixture was transferred into the volumetric flask and mixed with 1 M nitric acid. The ash was diluted with lanthanum chloride in order to release calcium. The quantification of the calcium was analyzed at the wavelength of 422.7 nm.

### 2.4. DNA Extraction and Assessment of DNA Quality

After verifying the integrity of the packages, the tuna cans were surface-cleaned and stored under controlled conditions in the laboratory. As a filling medium, oil, spice, and sauces were removed from tissues by using a sterile filter paper to press and blot prior to the DNA extraction. The tuna muscle (∼15 g) from each sample was placed in a sterile 50-mL falcon tube using sterile forceps. DNA extraction from 70 mg of tuna tissue was subsequently carried out by using Qiagen’s DNeasy Blood and Tissue Kit with the Spin Column Protocol (Qiagen, Valencia, CA, USA), according to the manufacturer’s protocol with slight modifications. Briefly, the tuna tissue was homogenized in ATL buffer (250 µL) using a tissue lyser (Tissue Lyser II, Qiagen, Valencia, CA, USA). Twenty μL proteinase K were added, mixed, and incubated at 56 °C until the tissue was completely lysed. The lysed solution was centrifuged at 12,000× *g* for 30 s, then the pellet was washed with AW1 (650 μL) and AW2 (500 μL) buffer within the QIAamp spin column. Finally, the DNA was eluted twice by adding a preheated Buffer AE (25 µL) at 37 °C to increase the yield. The quantity and quality of the genomic DNA were determined using a NanoDrop ND-1000 Spectrophotometer (ThermoFisher, Waltham, MA, USA) by measuring the absorbance at 230, 260, and 280 nm. The impact of the filling medium on the extraction success, and the DNA yield was also determined. The DNA quality parameters are important for the further amplification process and determination of parvalbumin in the canned tuna process.

### 2.5. Species Identification within DNA Barcoding and DNA Mini Barcoding

DNA barcoding and mini barcoding approaches were applied within the COI and 12 S gene regions, respectively, for the species authentication of the canned tuna (Table 2). Prior to a sanger sequencing, a cleaning process followed this; 5 µL of amplicon and 2 µL of ExoSAP-IT™ (Applied Biosystems™, Waltham, MA, USA) were mixed and a thermal process (37 °C for 30 min, at 80 °C for 15 min) was applied using a SimpliAmp™ thermal cycler (Applied Biosystems™, Waltham, MA, USA). The PCR reactions were then performed as follows: they contained 2 μL of template DNA, 10 μL Master Mix (Thermo Scientific™ Maxima SYBR Green/ROX qPCR Master Mix (2×)), 2 μL of each primer, and 6 μL DNA-free water. The PCR reactions for both primers were performed with same protocols using a SimpliAmp™ (Applied Biosystems™, Waltham, MA, USA) PCR System. The amplified fragments were directly sequenced using an ABI 3130xl Genetic Analyzer (Applied Biosystems, Waltham, MA, USA) to control the correct species. For this purpose, 5 µL of amplicon and 2 µL of ExoSAP-IT™ (Applied Biosystems™, Waltham, MA, USA) were mixed in a strip tube, and cycled in a SimpliAmp™ (Applied Biosystems™, Waltham, MA, USA), thermally, at 37 °C for 30 min, and at 80 °C for 15 min. Samples were subjected cycling for: 1 min at 96 °C; 10 s at 96 °C; 5 s at 50 °C; 4 mins at 60 °C, for 25 cycles), and were incubated at 4 °C in a SimpliAmp™ (Applied Biosystems™, Waltham, MA, USA) thermal cycler. After the sequence, a PCR study was completed, and the samples were subjected to physical cleaning. The samples were analyzed using the 3130xl Genetic Analyzer (Applied Biosystems^®^, Waltham, MA, USA). After the analysis of the contigs, consensus sequences were exported in a Fasta format for each sample for the data analysis. The generated sequences were all subjected to a BLASTn analysis at NCBI (https://blast.ncbi.nlm.nih.gov/Blast.cgi) (accessed on 20 May 2022) to identify the species. Sequence alignments were performed with CLUSTAL W within the MEGA X version (as per Kumar et al.) [26].

### 2.6. Parvalbumin Detection by Real-Time PCR

Libraries were prepared with a parvalbumin-specific primer (GenBank accession No. AB375265.1) by following the procedure that is described by Guo et al. [23], using the target sequence 664 bp region of the tuna allergen-specific gene sequence which was aligned by using the Clustal Omega program. A real-time PCR was performed in a StepOnePlus Real-Time PCR System (Applied Biosystems, Waltham, MA, USA). The final reaction volume was 20 μL, containing 10 μL Maxima SYBR Green/ROX qPCR Master Mix (2×) (Thermo Scientific™ 1 μM each of the forward and reverse primers, and 20 ng of the genomic DNA). The real-time PCR was performed as previously described by Guo [29], as follows: initial denaturation at 95 °C for 2 min, 35 cycles of denaturation at 95 °C for 10 s, and annealing at 55 °C for 30 s. There was an extension at 72 °C for 7 min. Fluorescent signals were read at the end of the annealing step during each cycle. After the final PCR cycle, a melt curve was carried out by means of employing heating amplicons from 65 °C to 95 °C with an increment of 5 °C/s for the determination of the differences in the melting temperature, and additional/abnormal peak formation, and the results were analyzed using the software to identify the Tm of the PCR product. Melt curves were converted into melting peaks by plotting the negative derivatives of the relative fluorescence (RFU) versus the temperature (−d(RFU)/dT), which can be visualized as peaks characterizing the Tm of the double-stranded DNA complexes. Standard curves for the real-time PCR were generated using the cycle threshold (Ct) value which was obtained from 10-fold serial dilutions of canned tuna genomic DNA, creating the standard curves for the RT-PCR. The efficiency (10(−1/slope − 1)) of each reaction was determined from the slope of the standard curve (Ct), as indicated in the Applied Biosystems. For the determination of the absolute sensitivity, the enhanced fluorescent SYBR-GREEN dye was used for the determination of parvalbumin in the extracted DNA from the canned tuna samples. This was due to DNA binding dyes facilitating the acquisition of high fluorescence upon intercalation into double-stranded DNA (dsDNA) or by binding to the minor grooves of dsDNA.

### 2.7. Gel Electrophoresis Analysis

Following the RT-PCR amplification, which yielded the PCR products of the parvalbumin response gene, the allergen responsible for the protein was confirmed by gel electrophoresis (2% agarose gel in Tris-Sodium acetate-EDTA (TAE)) buffer containing 1× SYBR Safe DNA Gel Stain (Life Technologies, Carlsbad, CA, USA) at 100 V. Finally, the gel was visualized using a UV light imaging analyzer (Vilber Lourmat Peqlab FUSION SL gel documentation system, Vilber Lourmat, Deutschland GmbH, Eberhardzell, Deutschland). The low ladder (M1031) (Dongsheng Biotech, Guangzhou, China) was used as a reference.

### 2.8. Statistical Analysis

All analyses were carried out in triplicate and the results are given as average ± standard deviation. The data was analyzed using SPSS 22.0 software (IBM SPSS Statistics, Chicago, IL, USA). An ANOVA and a Tukey post-hoc test at *p* values of 0.05 were run to clarify significant variance among the canned tuna groups.

## 3. Results

### 3.1. pH and Calcium Content Differences among Canned Tuna Groups

Different components were used as can-filling mediums in canned tuna which causes variation in the acidity of the main food items and this was seen during the thermal processing. The results related to the impact of the can-filling medium on twenty-nine canned tuna samples and filling mediums (liquid phase) are given in Table 3. The highest and lowest pH values were determined in olive oil-containing and sunflower oil/sauce-containing canned tuna groups (6.03 and 5.73, respectively). The lowest pH of sunflower oil-containing and different sauce-containing canned tuna groups could be a result of the low pH of tomatoes. The pH value of canned tuna containing sunflower/spice was found at 5.93.

There are significant differences determined among the canned tuna groups in terms of their calcium content. The highest calcium level was determined in the canned tuna with groups with sunflower oil and sauces, especially in the tomato sauce-containing canned tuna groups (171.96 mg/100 g). The lowest calcium level was determined in the canned tuna containing a brine solution (7.04 mg/100 g). These findings comply with the results that are reported by Alam et al. [30], who reported that the calcium levels of canned fish differ depending on the canned fish species, and the food matrices that were used as a filling medium, and the storage duration. Parvalbumin is a calcium-binding protein and the impact of calcium presence on parvalbumin has been noted by some research; Swoboda et al. [31] reported that through the removal of calcium from carp parvalbumin, the IgE binding capacity was reduced by 57%. Permyakov et al. [32] highlighted that depletion of calcium caused a structural change in the parvalbumin. De Magalhães et al. [33] indicated that the presence of EDTA in canned fish acts as a calcium chelator which leads to a 50% reduction in the parvalbumin’s IgE-reactivity in gilt-head seabream. Therefore, the relationship between parvalbumin and calcium presence is conducted by various research.

### 3.2. Yield and Quality of DNA

Can-filling mediums cause further limitations to the extraction of high-quality DNA from canned tuna samples due to the thermal conductivity and acidic properties of various filling medium ingredients. The DNA yield was calculated, based on the DNA concentration, initial tuna sample weight, and obtained the final volume. There were significant differences among the filling mediums in commercial canned tuna products (*p* < 0.05) in terms of the DNA yield (Table 4). While the general DNA yield of canned tuna differs from 2.00 to 26.2 ug/uL among canned tuna groups, some of the samples have extremely higher DNA yields which reached up to 1682 ug/uL. These results are valuable for understanding the importance of the food matrix on the extracted DNA yield. Since the same amount of tuna flesh was used for DNA extraction and the same protocol was applied to all groups, the higher DNA yield caused some concerns about sample processing and the potential risks in the process. Even though the tuna flesh was filtered and all the matrix components were removed, the filling mediums had impacted the tuna flesh pH and therefore, the DNA degradation of the sample. The lowest DNA yield (2.00 ug/uL) was obtained from the canned tuna with a relatively higher pH (6.2) among the sampling groups.

Another important quality parameter is the rate of 260/230 which indicates the presence of chemical contamination in the extracted DNA and causes challenges in the further analysis steps. The significant differences were determined among canned tuna groups with various can-filling mediums in terms of A260/A230 (*p* < 0.05) (Table 4). The purity of the extracted DNA is another key factor in the amplifiability of DNA which can be used for food traceability and the detectability of any compounds. The optimal range for this ratio is set at 1.8–2.0. While there is no correlation between the can-filling mediums and the purity of DNA, the canned tuna groups that had the optimal purity were found with a pH that was higher than 5.9 (Table 4). The pH of the different compounds that were used in the can-filling mediums impacts the acidity of the food, which can limit the detection of the higher purity of DNA.

### 3.3. Species Authentication of Canned Tuna

As mentioned above, the term, tuna, is commonly used for different members of the Scomboridae family. The results of the DNA barcoding and mini-barcoding are shown in Table 5. All 28 successfully sequenced samples were identified to the species level, with the top sequence matches for 28 of these samples showing >90% identity and ≥92% query coverage with BLAST. Six of the total twenty-nine samples failed within the DNA full barcoding test. Five of six failed samples were identified by the DNA mini-barcoding. These results revealed that the achievements of DNA mini barcoding were higher than those of the DNA barcoding. These findings are in accordance with Shokralla et al. [23] who highlighted the qualification and quantification of DNA mini barcoding for intraspecific species identification.

The identification of intraspecific differentiation showed that while 28 were successfully amplified in the canned tuna sample, there were the following differences among these, 11: Skipjack tuna (K. pelamis), nine: Yellowfin tuna (T. albacares), four: Auxis thazard (Frigate tuna), two: Albacore (Thunnus alalunga), and two: Auxis thazard (Frigate tuna).

### 3.4. Parvalbumin Gene Detection as a Marker for Fish Allergens in Canned Tuna 

**Figure 1:**
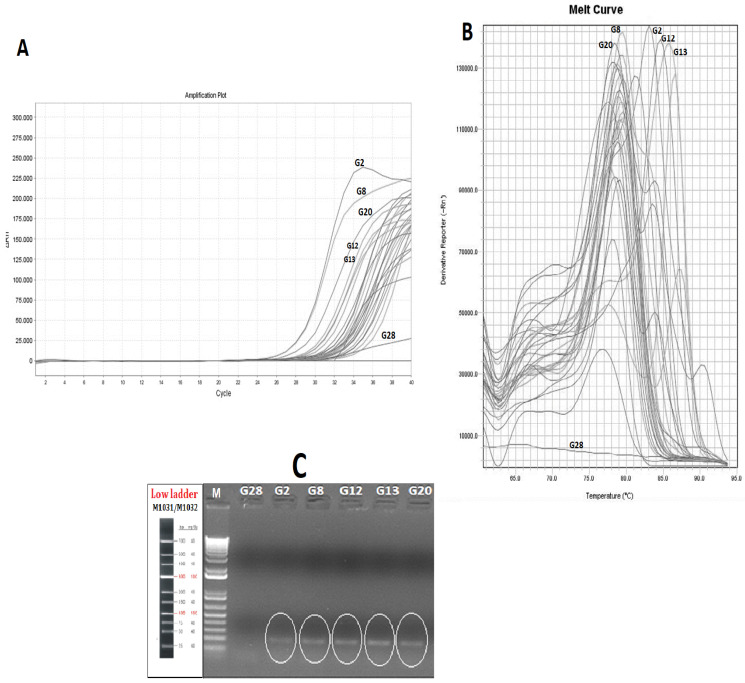
Cycle threshold (Ct) (**A**); Melting curves (**B**) of parvalbumin-encoded gene amplification by RT-PCR (**C**); confirmation of parvalbumin in canned tuna groups on gel electrophoresis (G28: Non-amplified, as negative control, Canned tuna in Sunflower oil; G2: Canned tuna in sunflower oil/tomato sauce; G8: Canned tuna in sunflower oil/canola oil; G12: Canned tuna in sunflower oil; G13: Canned tuna in sunflower oil/spiced; G20: Canned tuna in sunflower oil/tomato sauce).

In this study, fish allergens-coding sequences have been used as targets to develop a reliable real time-PCR protocol to detect tuna allergens in canned tuna with different can-filling mediums. While the presence of the allergen in food products is determined by protein-based methods, traditionally, the encoding of specific allergen genes in food matrices is accepted as a more reliable method, owing to the DNA’s resistance to heat and it being highly processing. The analyzing of the allergen proteins by using protein-based methods has been accepted as most traditional approach. Allergen–coding gene region detection has applied as an alternative and reliable approach for the detection of food-borne allergens. There are many research studies that have reported the detection of allergens by using PCR-based applications such as loop-mediated and RT-PCR without any protein-based method confirmation. For instance, Sanchiz et al. [34] reported that they used “Ara h 6” as a peanut allergen-coding region for the determination of peanut allergens that are impacted by different processing methods. Additionally, Toricelli et al. [35] highlighted the achievements of the 2S albumin gene, performed by real-time PCR, for sesame, pistachio, and macadamia detection in commercial food samples. The allergen-encoding amplification results of the canned tuna groups are given in Figure 1A. Except for a sample group (G28), all the canned tuna samples were amplified successfully. The RT-PCR results revealed that the mean of the Ct (cycle that crosses the threshold) value is around 30.020. While there is no direct positive or negative correlation between the allergen content and Ct values, there are some differences among the groups that indicate the alteration in the allergen response gene. In this research, the amplification specificity of the PCR products was confirmed by the post-PCR melt curve analysis which is considered to be a robust tool for the molecular diagnostic of food-borne allergens. The melt curve analysis of the real-time PCR assay showed that the Tm values of the canned tuna differed from 76.8 to 85.8 °C. Out of 29 canned tuna samples, five groups were found to be allergen-positive (Figure 1B). The presence of parvalbumin was determined in the canned tuna containing tomato sauce (G2 and G20) with 86.4 and 84.7 Tm., respectively. Other canned tuna groups that have relatively higher melt curve values are G8, G12, and G13 with 85.85, 81.33, and 83.10 Tm values, respectively. Interestingly, out of five parvalbumin positive groups, two of them (G12 and G13 that have sunflower oil and sunflower oil/spiced can-filling medium, respectively) were obtained from the same brand and the initial DNA yield of these groups was found to be significantly higher than of the other groups (Table 2). These results raise a concern about the canning process of these two samples which can be a result of the lack of a correct and sufficient sterilization process in terms of elements that are related to time or heat. Another allergen-positive group was G8, representing canned tuna with an oil mixture consisting of sunflower oil and canola oil (Figure 1B).

**Figure 2:**
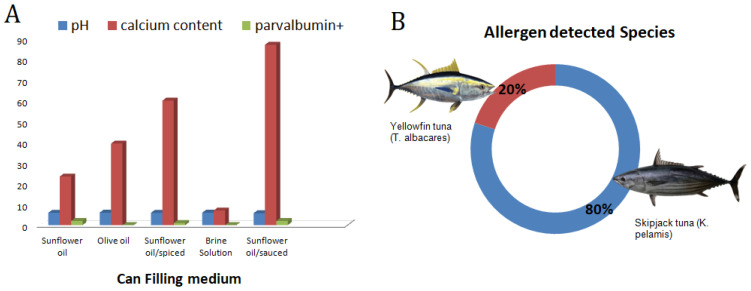
(**A**) Can-filling medium impact on pH, calcium level, and allergen response. (**B**) species distribution impact on the fish allergen.

From the 29 analyzed samples, five allergen-positive products were determined (17.2%) and these were varied as the can-filling mediums of the fish allergen-positive groups were: two with tomato-sauce, one that was spiced, one that contained canola oil, and one that contained sunflower oil. Since the tomato, the canola oil, or any of the spices cannot contain parvalbumin proteins, these results can be explained by these ingredients’ impacts on the solubility and structure of them by the process of them binding to the present allergens in the canned tuna. According to previously reported research that was related to impact of complex food matrices on the food-borne allergens. Kenk et al. [36] and Villa et al. [37] reported that lupine was an allergenic food that was detected and impacted by food matrices. Costa et al. [38] also reported on three allergen-encoding genes that were used for the detection of cashew nuts in complex matrices such as wheat, dough, and biscuits. They reported that the detectability of the target genes were impacted by food matrices. The specificity of RT-PCR was confirmed by gel electrophoresis. The results of the agarose gel electrophoresis of the target fish allergen in the canned tuna groups which are found to be positive according to melt curve peak are shown in Figure 1B. The presence of the allergen response sequencing results of the amplicons was further confirmed by the gel electrophoresis. In addition to the allergen-positive groups, one of the non-amplified (G28) groups was used as a negative control in the agarose electrophoresis and no bands were observed in the relevant tuna group (Figure 1C). The absence of a band in the non-amplified group indicated the specificity of the RT-PCR and agarose electrophoresis analyses. We performed the gel electrophoresis, following the RT-PCR, to confirm the allergen coding genes’ presence in the tested sample, and the same approach was also applied by Li et al. [39] for the detection of allergens in milk pudding, cheese, and shrimp ball by using RT-PCR-based methods. These researchers also mentioned the difficulties of the isolation of the DNA in complex food matrices. The factors impact on the presence of the fish allergen-encoding genes are summarized in Figure 2A: Can filling medium impacts, Figure 2B: Tuna sub-species impact on the fish allergen.

## 4. Conclusions

In this study, the potential reasons for the presence of one of the most important fish allergens, parvalbumin, in commercial, canned tuna products from 13 different brands, with the use of 29 samples, were assessed. The impacts of the pH and calcium content of the different can-filling mediums on the allergen levels were also evaluated, as well as the filling medium impact on the DNA-based methods for allergen detection and species identification. The quality parameters such as yield, purity, and presence of any contaminants on DNA were evaluated.

Out of 29 parvalbumin-suspected samples, five groups were found as positive, and two of them had relatively higher DNA yield which caused suspicion about the canning process. The parvalbumin encoding gene was determined in all tomato sauce-containing canned tuna groups. Interestingly, the parvalbumin-encoding gene was not detected in any of the canned tuna samples that contained olive oil. The relatively higher calcium contents of commercial tuna caused the presence of an allergen-coding gene.

DNA barcoding and DNA mini barcoding approaches were applied for the determination of the cryptic species and the potential sub-species that are able to carry allergen-encoding genes in commercially canned tuna.

According to the obtained results of the amplicon, specified by the melt curve analyses and confirmed by the gel electrophoresis, in terms of tuna species’ impact on the fish allergen gene, four out of the five samples were amplified successfully with a fish allergen marker, and these were determined as Skipjack tuna (*K. pelamis*) and one of them was Yellowfin tuna (*T. albacares*).

The results revealed that the fish allergen mechanism is impacted by internal and external factors. Although the findings of this research can contribute to the understanding of the effects of the selected factors (fish type, can filling, pH, and calcium content) on the parvalbumin level in commercially canned tuna, the author strongly believe there are still some gaps in the knowledge that is related to allergen occurrence mechanisms and stability in canned tuna, such as the controlling of raw materials before being processing. Therefore, further research will be focused on the detection of allergens and a better understanding of the stability and mechanisms of food-borne allergens in raw fish.

## Figures and Tables

**Table 1:** Sample descriptions of canned tuna.

Sample ID	Commercial Product Type	Canning Filing Medium	Brand	Exp. Date
1	Canned tuna	Sunflower oil/vegetable seasoning	Brand 1	21 December 2024
2	Tomato sauced tuna	Sunflower oil/tomato sauce	Brand 1	19 April 2025
3	Mustard sauced tuna	Sunflower oil/mustard sauce	Brand 1	15 June 2025
4	Canned tuna	Olive oil	Brand 1	20 October 2024
5	Canned tuna	Sunflower oil	Brand 1	19 October 2025
6	Canned tuna	Olive oil	Brand 2	19 September 2025
7	Canned tuna	Sunflower oil	Brand 2	11 April 2024
8	Canned tuna	Sunflower oil/canola oil	Brand 2	11 January 2025
9	Tuna salad	Sunflower oil/olive oil/corn	Brand 2	05 November 2024
10	Tuna salad	Sunflower oil/olive oil/beans	Brand 2	05 September 2024
11	Canned tuna	Sunflower oil	Brand 2	14 February 2025
12	Canned tuna	Sunflower oil	Brand 3	15 November 2024
13	Spiced canned tuna	Sunflower oil/Spiced	Brand 3	22 March 2025
14	Canned tuna	Brine solution	Brand 4	12 February 2025
15	Canned tuna	Sunflower oil	Brand 5	18 April 2025
16	Canned tuna	Brine solution	Brand 6	20 July 2025
17	Canned tuna	Sunflower oil	Brand 7	19 December 2024
18	Canned tuna	Sunflower oil	Brand 8	28 April 2025
19	Canned tuna	Sunflower oil	Brand 9	04 March 2025
20	Tomato sauced canned tuna	Sunflower oil/tomato sauce	Brand 10	29 December 2024
21	Olive oiled canned tuna	Olive oil	Brand 5	26 December 2024
22	Spiced canned tuna	Sunflower oil/Spiced	Brand 2	15 September 2024
23	Canned tuna	Sunflower oil	Brand 6	24 December 2024
24	Canned tuna	Olive oil	Brand 9	14 February 2025
25	Canned tuna	Sunflower oil	Brand 11	26 January 2025
26	Canned tuna	Sunflower oil	Brand 12	30 January 2025
27	Spiced canned tuna	Spiced/sunflower oil	Brand 12	19 December 2024
28	Canned tuna	Sunflower oil	Brand 13	27 February 2025
29	Canned tuna	Sunflower oil	Brand 6	17 March 2025

**Table 2:** Primer sets that were used for tuna species identification assessed in this study.

Locus	Code	Sequence (5′–3′)	Amplicon Base Pair (bp)	Reference
COI F	FishF2	TCGACTAATCATAAAGATATCGGCAC	655	Ward et al. [27]
COI R	FishR2	ACTTCAGGGTGACCGAAGAATCAGAA		
Teleo F	L848	ACACCGCCCGTCACTCT	100	Valentini et al. [28]
Teleo R	H1913	CTTCCGGTACACTTACCATG		

**Table 3:** pH and calcium content of canned tuna groups.

Sample ID	pH of Filling Medium	pH of Tuna Meat	Calcium Content (mg/100 mg)
1	5.6 ^f^	5.9 ^c^	62.40 ^d^
2	5.1 ^a^	5.4 ^a^	165.90 ^e^
3	5.2 ^b^	5.4 ^a^	74.45 ^d^
4	5.3 ^c^	5.8 ^b^	15.41 ^b^
5	5.2 ^b^	5.8 ^b^	14.35 ^b^
6	6.1 ^h^	6.5 ^e^	22.45 ^b^
7	5.2 ^b^	5.9 ^c^	34.15 ^c^
8	5.2 ^b^	5.8 ^b^	16.21 ^b^
9	5.5 ^e^	5.9 ^c^	24.15 ^b^
10	5.5 ^e^	5.9 ^c^	16.34 ^c^
11	5.3 ^c^	5.8 ^b^	21.25 ^b^
12	5.4 ^d^	5.8 ^b^	15.13 ^b^
13	5.3 ^c^	5.8 ^b^	54.05 ^d^
14	5.5 ^e^	5.9 ^c^	7.16 ^a^
15	5.4 ^d^	5.8 ^b^	29.45 ^b^
16	5.9 ^g^	6.1 ^d^	6.92 ^a^
17	5.4 ^d^	5.8 ^b^	23.44 ^b^
18	5.3 ^c^	5.9 ^c^	16.32 ^b^
19	5.8 ^g^	6.2	12.02 ^b^
20	5.6 ^f^	5.9 ^c^	178.02 ^e^
21	5.8 ^g^	6.0 ^d^	16.54 ^d^
22	5.7 ^f^	5.9 ^c^	63.42 ^d^
23	5.6 ^f^	5.9 ^c^	33.06 ^c^
24	5.6 ^f^	5.8 ^b^	43.80 ^c^
25	5.8 ^g^	6.1 ^d^	19.17 ^b^
26	5.9 ^g^	6.1 ^d^	27.12 ^b^
27	5.7 ^f^	6.1 ^d^	62.42 ^d^
28	6.1 ^h^	6.4 ^e^	12.9 ^b^
29	5.9 ^g^	6.1 ^d^	19.90 ^b^

Data are expressed as mean value ± standard deviation of triplicates. Values followed by different letters (^a–h^) indicate significant differences (*p* < 0.05).

**Table 4:** Quality differences among the tuna groups.

Sample ID	DNA Yield (ug/uL)	Purity (A260/A280)	Chemical Contamination (A260/A230)
1	5.9 ^a^	1.24 ^d^	1.09 ^e^
2	11.4 ^b^	1.23 ^d^	0.56 ^d^
3	32 ^c^	1.71 ^f^	1.03 ^e^
4	15.4 ^b^	1 ^c^	0.47 ^c^
5	10.6 ^b^	1.33 ^d^	0.44 ^c^
6	2.6 ^a^	0.85 ^b^	−0.39 ^b^
7	4 ^a^	0.89 ^b^	0.44 ^c^
8	6.2 ^b^	1.26 ^d^	0.41 ^c^
9	4.5 ^a^	1.2 ^d^	0.78 ^d^
10	56.3 ^c^	2.05	1.42 ^f^
11	11.5 ^b^	1.09 ^c^	0.43 ^c^
12	530.3 ^e^	2 ^g^	2.07 ^f^
13	1682 ^f^	2.11 ^g^	2.24 ^f^
14	3.6 ^a^	1.32 ^d^	−1.67 ^b^
15	9.5 ^b^	1.6 ^e^	−13.64 ^a^
16	26.2	1.80 ^f^	1.24 ^e^
17	3.4 ^a^	1.49 ^d^	−0.37 ^b^
18	6.2 ^a^	1.08 ^c^	1.21 ^e^
19	2.0 ^a^	1.85 ^f^	−0.23 ^b^
20	240.1 ^d^	1.93 ^f^	2.52
21	280.9 ^d^	2.1 ^g^	2.44 ^f^
22	3.4 ^a^	−0.78 ^a^	−1.21 ^b^
23	13.0 ^b^	1.7 ^f^	1.87 ^e^
24	3.8 ^a^	1.54 ^e^	−0.76 ^b^
25	13.7 ^b^	1.07 ^c^	0.32 ^c^
26	7.1 ^b^	1.32 ^d^	1.1 ^e^
27	2.4 ^a^	1.05 ^c^	0.98 ^e^
28	2.9 ^a^	1.28 ^d^	0.56 ^d^
29	9.9 ^b^	1.39 ^d^	1.4 ^e^

Values followed by different letters (^a–h^) indicate significant differences (*p* < 0.05).

**Table 5:** Identified species by DNA-barcoding and DNA Mini-barcoding.

Sample ID *^a^*	DNA Full Length Barcoding	DNA Mini Barcoding
1	Skipjack tuna (*K. pelamis*)	Skipjack tuna (*K. pelamis*)
2	Skipjack tuna (*K. pelamis*)	Skipjack tuna (*K. pelamis*)
3	Yellowfin tuna (*T. albacares*)	Yellowfin tuna (*T. albacares*)
4	Skipjack tuna (*K. pelamis*)	Skipjack tuna (*K. pelamis*)
5	Bigeye tuna (*T. obesus*)	Bigeye tuna (*T. obesus*)
6	Bigeye tuna (*T. obesus*)	Bigeye tuna (*T. obesus*)
7	Skipjack tuna (*K. pelamis*)	Skipjack tuna (*K. pelamis*)
8	Skipjack tuna (*K. pelamis*)	Skipjack tuna (*K. pelamis*)
9	Yellowfin tuna (*T. albacares*)	Yellowfin tuna (*T. albacares*)
10	Failed PCR	Auxis thazard *(Frigate tuna)*
11	Auxis thazard (Frigate tuna)	Auxis thazard *(Frigate tuna)*
12	Failed PCR	Yellowfin tuna (*T. albacares*)
13	Skipjack tuna (*K. pelamis*)	Skipjack tuna (*K. pelamis*)
14	Skipjack tuna (*K. pelamis*)	Skipjack tuna (*K. pelamis*)
15	Auxis thazard (Frigate tuna)	Auxis thazard *(Frigate tuna)*
16	Skipjack tuna (*K. pelamis*)	Skipjack tuna (*K. pelamis*)
17	Yellowfin tuna (*T. albacares*)	Yellowfin tuna (*T. albacares*)
18	Albacore (*Thunnus alalunga*)	Albacore (*Thunnus alalunga*)
19	Failed PCR	Auxis thazard *(Frigate tuna)*
20	Skipjack tuna (*K. pelamis*)	Skipjack tuna (*K. pelamis*)
21	Yellowfin tuna (*T. albacares*)	Yellowfin tuna (*T. albacares*)
22	Failed PCR	Albacore (*Thunnus alalunga*)
23	Yellowfin tuna (*T. albacares*)	Yellowfin tuna (*T. albacares*)
24	Skipjack tuna (*K. pelamis*)	Skipjack tuna (*K. pelamis*)
25	Failed PCR	Yellowfin tuna (*T. albacares*)
26	Skipjack tuna (K. pelamis)	Skipjack tuna (*K. pelamis*)
27	Failed PCR	Yellowfin tuna (*T. albacares*)
28	Failed PCR	Failed PCR
29	Yellowfin tuna (*T. albacares*)	Yellowfin tuna (*T. albacares*)

^*a*^ Top species match was ≥98% genetic similarity.
